# In Vitro Neutralisation of Rotavirus Infection by Two Broadly Specific Recombinant Monovalent Llama-Derived Antibody Fragments

**DOI:** 10.1371/journal.pone.0032949

**Published:** 2012-03-05

**Authors:** Farah Aladin, Alexandra W. C. Einerhand, Janneke Bouma, Sandra Bezemer, Pim Hermans, Danielle Wolvers, Kate Bellamy, Leon G. J. Frenken, Jim Gray, Miren Iturriza-Gómara

**Affiliations:** 1 Enteric Virus Unit, Centre for Infections, Health Protection Agency, London, United Kingdom; 2 Laboratory of Pediatrics, Erasmus MC-Sophia, Rotterdam, The Netherlands; 3 BAC B.V., Naarden, The Netherlands; 4 Unilever Research and Development Vlaardingen B.V., Vlaardingen, The Netherlands; 5 Unilever Research and Development Colworth House, Sharnbrook, United Kingdom; Tulane University, United States of America

## Abstract

Rotavirus is the main cause of viral gastroenteritis in young children. Therefore, the development of inexpensive antiviral products for the prevention and/or treatment of rotavirus disease remains a priority. Previously we have shown that a recombinant monovalent antibody fragment (referred to as Anti-Rotavirus Proteins or ARP1) derived from a heavy chain antibody of a llama immunised with rotavirus was able to neutralise rotavirus infection in a mouse model system. In the present work we investigated the specificity and neutralising activity of two llama antibody fragments, ARP1 and ARP3, against 13 cell culture adapted rotavirus strains of diverse genotypes. In addition, immunocapture electron microscopy (IEM) was performed to determine binding of ARP1 to clinical isolates and cell culture adapted strains. ARP1 and ARP3 were able to neutralise a broad variety of rotavirus serotypes/genotypes in vitro, and in addition, IEM showed specific binding to a variety of cell adapted strains as well as strains from clinical specimens. These results indicated that these molecules could potentially be used as immunoprophylactic and/or immunotherapeutic products for the prevention and/or treatment of infection of a broad range of clinically relevant rotavirus strains.

## Introduction

Rotavirus is a non-enveloped, icosahedral virus of the *Reoviridae* family containing a genome of 11 segments of double stranded RNA (dsRNA). Recently, it has been estimated that each year, rotavirus causes more than a 100 million episodes of gastroenteritis which results in 25 million clinic visits, 2 million hospitalizations, and more than 611,000 deaths in children below 5 years of age [1]. By 5 years of age, nearly every child worldwide will have had at least one episode of rotavirus gastroenteritis [2]. Children in developing countries account for 82% of rotavirus deaths. Therefore, rotavirus remains the most important cause of severe and life threatening viral gastroenteritis and dehydrating diarrhoea in young children worldwide [3], [4], [5].

Rotavirus replicates in mature enterocytes of the small intestine leading to a reduction of enterocyte-specific gene expression and an induction of virus gene expression and inflammatory mediators and is thought to be a multi-factorial process [6], [7] which include a reduction in epithelial surface area, replacement of mature enterocytes by immature cells, down regulation of genes involved in digestion and absorption of nutrients, salt and water, an osmotic effect resulting from incomplete absorption of carbohydrates from the intestinal lumen and the secretion of intestinal fluid and electrolytes through activation of the enteric nervous system (reviewed in [8], [9], [10], Despite the prevalence of rotavirus diarrhoeal disease and extensive studies in different animal models, rotavirus pathogenesis is still not completely understood.

Rotaviruses are currently divided into seven serotypes (Rotavirus A–G). They exhibit broad genetic and antigenic diversity due to reassortment among rotavirus strains and the accumulation of point mutations in the surface protein genes. Group A rotaviruses are the major human pathogens, and have been further categorised on the basis of the outer capsid proteins, VP4 (P-type) and VP7 (G-type), and the intermediate layer protein VP6 (subgroups [SG]). Currently, there are 35 P-types and 27 G-types [11], [12], [13], [14] and four VP6 SGs [15], [16] recognised. As well as showing different G and P types and a variety of combinations of these, there is also intratypic variation. The incidence and distribution of group A rotavirus genotypes varies between geographical areas during a rotavirus season, and from one season to the next [17]. Globally, G1P [8], G2P [4], G3P [8], G4P [8] and G9P [8] are the most common G and P types of rotavirus causing disease in humans. However, the introduction of molecular typing methods has revealed the existence of other G and P types such as G5, G6, G8, G10, G12, P [6] and P [11] causing infection in humans which have most likely emerged through zoonotic transmission. Zoonotic transmission and the ability of rotaviruses to reassort following double infections provide the potential for the emergence of novel strains [18].

Several oral, live-attenuated vaccines have been developed in recent years. Two of them have been licensed and are in use in several countries in universal vaccination programmes. There is, to date, no satisfactory therapeutic means for controlling rotavirus disease, and alternative therapies are thus needed urgently. Also, prophylactic measures, in particular in a high risk setting (for example, outbreaks, the immunocompromised, etc) may be a useful addition to current rotavirus prevention strategies. The usefulness of any such treatment will be determined to a great extent on their ability to be effective against the broad spectrum of rotavirus types commonly circulating in the population worldwide.

Previously we have shown that specific antibody fragments derived from llama heavy chain antibodies (VHH fragments) can be obtained against different types of antigens [19]. Furthermore, by using modern biotechnology, these fragments can be produced in bakers yeast, *Saccharomyces cerevisiae*, in a cost effective way [20]. It has been demonstrated that monovalent VHH binding domains can neutralise bacteriophages [21]. Previously, we described the production of anti-rotavirus VHH fragments after immunising a llama with the rhesus rotavirus (RRV) [22]. It was demonstrated that one of these anti-rotavirus VHH fragments (VHH1, now referred to as Anti-Rotavirus Protein 1 or ARP1) was able to neutralise RRV in an mouse model system [22]. Furthermore, in a recent human intervention study ARP1 has been shown to reduce the stool output in young children with rotavirus diarrhoea with about 50% (Sarker *et al*, submitted).

This study describes the ability of ARP1 and ARP3 (derived in the same manner as ARP1) fragments to bind and neutralise rotaviruses of different genotypes, including those genotypes found with the highest incidences in cases of infantile diarrhoea worldwide. The knowledge obtained from this work may be useful for the development of compounds able to prevent rotavirus diarrhoea in young children.

## Materials and Methods

### Rotavirus strains

For the *in vitro* neutralization studies, cell culture adapted rotavirus strains WI61 (G9P [8]) and DS-1 (G2P [4]) were obtained from the ATCC. The human rotavirus strain Wa (G1P [8]), one strain of the rhesus rotavirus strain RRV (G3P [3]) and the simian rotavirus strain SA-11 (G3P [1]) were kindly provided by Dr. M. Koopmans (RIVM, Bilthoven, The Netherlands). Cell culture adapted strains ST-3 (G4P [6]), 69M (G8P [10]), RV4 (G1P [8]), F45 (G9P [8]), Va70 (G4P [8]) and P (G3P [8]) were kindly provided by Dr C. Kirkwood (Murdoch Children's Research Institute, Melbourne, Australia), and a second strain of RRV was obtained from Dr Harold Marcotte of the Karolinska Institute, Stockholm, Sweden. These strains were cultured according to the methods previously described [23]. For the IEM studies, cell culture adapted and human stool samples containing G1P [8], G2P [4], G3P [8], G4P [8], G9P [6], G9P [8], G10P [11] and G12P [9] rotavirus strains were used.

### Antibody fragments

Llama derived ARP1 and ARP3 raised against a G3P [3] rotavirus strain were obtained as described in Vaart *et al*. [22]. A control fragment from a llama immunized with the hapten antigen azodye RR6 coupled to BSA, VHH R2, was used throughout [20]. All 3 fragments were produced in yeast as previously described [20] and purified from yeast culture medium by ion exchange chromatography. In brief, the yeast culture medium was diluted 5 times in 25 mM sodium acetate buffer pH 4.5 (Sigma, Zwijndrecht, Netherlands) and loaded on a 10 ml Sp-Sepharose FF column (GE Healthcare, Little Chalfont, UK). Unbound material was removed by washing with 25 mM sodium acetate pH 4.5. Bound llama fragment was eluted with 40 mM Na_2_HPO_4_ pH 12 (Sigma, Zwijndrecht, Netherlands). The eluted fraction was brought to the biotinylation buffer (50 mM Ca_2_CO_3_ pH 8) using a PD10 column (GE Healthcare, Little Chalfont, UK).

ARP1, ARP3 and VHH R2 were biotinylated by adding NHS-biotin (N-Hydroxysuccinimidobiotin in DMSO, Sigma, Zwijndrecht, Netherlands) to the llama fragments in a molar ratio of 20∶1 (NHS-biotin : llama fragment). Unbound biotin was removed by dialysis against PBS after incubating on a rotary mixer for 2 hours at room temperature.

### Rotavirus neutralisation studies

The rotavirus neutralisation studies were performed independently in two laboratories, the Laborotory Pediatrics, Erasmus MC-Sophia, Rotterdam, The Netherlands, and The Enteric Virus Unit, Virus Reference Department, Health Protection Agency, London, UK.

At the Laboratory of Paediatrics, Erasmus MC, CaCo-2 (ATCC, HTB-37) or MA104 (ATCC, CRL-2378.1) cells were maintained in Dulbecco's Modified Eagle's Medium (DMEM, GibcoBRL, Paisley, Scotland) containing 10% (v/v) foetal calf serum (FBS, Integro, Dieren, The Netherlands), 100 U/ml Penicillin, 100 µg/ml Streptomycin and 1% (v/v) non-essential amino acids (BioWhittacker, Verviers, Belgium) at 37°C and in an atmosphere of 5% CO_2_-air.

To test the neutralising activity of the llama antibody fragment ARP1 and a control antibody (VHH R2), 1.5×10^4^ CaCo-2 cells were plated on heavy Teflon coated microscope slides (∅ 7 mm, Cell-line/Erie Scientific, Portsmouth, NH) as described previously [23]. Cells were rinsed 3 times with culture medium in the absence of FBS (DMEM-FCS) and incubated with different concentrations of the llama antibodies for 1 hour at 37°C prior to infection. Simultaneously, rotavirus was treated for 1 hour at 37°C with 10 µg/ml trypsin (Sigma, Zwijndrecht, Netherlands) diluted in DMEM-FCS. Subsequently, CaCo-2 or MA104 cells were inoculated with 100 fluorescent focus forming units (fffu) of rotavirus in absence or presence of decreasing concentrations of the llama antibodies (10 µg/ml-0.16 µg/ml in doubling dilutions. At 15 hours post-inoculation (p.i.), cells were fixed in ice-cold methanol at −20°C for 10 minutes and stored in phosphate buffered saline (PBS) pH 7.2. Infectivity was determined by an indirect immunofluorescence assay (IFA). The methanol-fixed cells were incubated for 90 minutes at room temperature with the polyclonal rabbit anti-rotavirus serum (K3ppIV, kindly provided by Dr. M. Koopmans, RIVM, Bilthoven, The Netherlands) diluted in PBS (1∶1600), rinsed four times with PBS, and stained for 1 hour with goat anti-rabbit Texas Red conjugated IgG (Jackson ImmunoResearch Laboratories Inc., West Grove, PA) diluted in PBS (1∶300). Finally, cells were washed extensively and mounted in Mowiol (Calbiochem, San Diego, CA) containing 2.5% w/v DABCO (1,4-diazabicyclo[2.2.2]octane) and 0.5 µg/ml DAPI (4′,6-diamidino-2-phenylindole dihydrochloride∶hydrate; Sigma-Aldrich, Zwijndrecht, Netherlands). Fluorescence was viewed with a Nikon Eclipse E800 microscope. The number of infected cells in the antibody-treated and control cells was expressed as a percentage of the average number of infected cells in the control cell cultures. Each titration experiment was performed at least twice.

At the Enteric Virus Unit, Health Protection Agency, London, UK, MA104 (ATCC, CRL-2378.1) cells were maintained in MEM (Life Technologies, Paisley, UK) supplemented with 10% foetal calf serum (Life Technologies, Paisley, UK) and gentamicin (Life Technologies, Paisley, UK) (50 mg/L). One hundred microliters of MA104 cells at a concentration of 1×10^5^ cells/ml were seeded onto 96-well cell culture plates (Cellstar, Greiner, Gloucestershire, UK) and incubated at 37°C, in an atmosphere of 5% CO_2_-air until cells were confluent (24–36 hours). Media was replaced with 100 µl serum-free media the day before infection and the cells were incubated overnight at 37°C in 5% CO_2_-air. Rotavirus strains were activated with porcine-trypsin (Sigma, Dorset, UK) at 5 µg/ml by incubation at 37°C for 30 minutes prior to infection. Trypsin-activated virus was diluted to 100 fffu per 100 µl (1×10^3^ fffu/ml) in a total volume of 5 ml serum-free media. A total of 100 µl (or 100 fffu) of activated virus was mixed with 100 µl of ARP1, ARP3 or VHH R2 antibodies in serial twofold dilutions in a separate dilution plate to give final antibody concentrations from 10 µg/ml to 0.16 µg/ml and incubated at 37°C for 1 hour. Experiments were performed in duplicate, and a control with no antibody was included in duplicate in each experiment. After removing media from the 96-well plate containing the MA104 cells, 200 µl of virus/VHH mix were added to the cells, the plates were sealed and centrifuged at 1000×g for 20 minutes. The plate-seal was removed and the inoculated cells were incubated overnight at 37°C in 5% CO_2_-air. Rotavirus infected cells were detected by immuno-fluorescence (IF). Cells were washed with 300 µl of PBS after removal of the inoculum and then fixed with methanol (VWR, West Sussex, UK) before air drying. A total of 100 µl/well of an anti-VP6 rotavirus monoclonal antibody (AMS Biotechnology, Abingdon, UK); diluted 1∶200 in PBS) was added and incubated at 37°C for 30 minutes. Cells were washed 3 times with PBS-Tween20 (0.05%) before 100 µl of a rabbit anti-mouse IgG FITC (Dako, Ely, UK) diluted 1∶20 in PBS with 0.005% Evans Blue (Euroimmun, Pontypool, UK) as a counterstain was added. Plates were incubated 30 minutes at 37°C followed by 3 washes with PBS-Tween20. Finally, cells were allowed to air dry and 50 µl of 10% glycerol (Sigma, Dorset, UK) in saline were added to prevent desiccation. Cells were visualised using an epifluorescence inverted microscope (Nikon Eclipse) and the number of fluorescent foci recorded. Experiments were conducted on at least 2 separate occasions for each strain and antibody concentration, and the antibody concentration needed to reduce the number of fluorescent foci by 50% was calculated for each antibody/rotavirus strain tested

### SDS-PAGE and Western blotting

Five millilitres of cell culture grown rotavirus was clarified by centrifugation at 1000×g, 10 minutes, the supernatant ultracentrifuged at 48,000×g, 45 minutes (Optima L-100 XP Ultracentrifuge, Beckman Coulter, USA) and the virus resuspended in 100 µl MEM (Life Technologies, Paisley, UK). Recombinant VP6 and VP7, expressed in a baculovirus/insect cell expression system and lyophilised (in house and in collaboration with Baylor College of Medicine, Houston, TX) were resuspended to 1 µg/ml each. Uninfected MA104 cells (used to propagate the virus) were included as a negative control. A total of 6.25 µl 4× NuPage LDS Sample Buffer (Life Technologies, Paisley, UK) and 2.5 µl 10× NuPage Reducing Agent (Life Technologies, Paisley, UK) were added to 16.5 µl sample, and incubated at 70°C, 10 minutes. Twenty five microlitres of denatured protein sample was separated on a 4–12% SDS-PAGE gel (Life Technologies, Paisley UK) at 100 V, 10 minutes then 150 V, 1 hour in 1× NuPage MOPS Running Buffer (Life Technologies, Paisley, UK). Proteins were transferred from the gel to a nitrocellulose membrane (GE Healthcare Life Sciences, Buckinghamshire, UK) in 1× NuPage Transfer Buffer (Life Technologies, Paisley, UK) with NuPage Antioxidant (Life Technologies, Paisley, UK) and 10% methanol (VWR, West Sussex, UK) for 1 hour at 30 V.

For the Western Blotting, the membrane was blocked in 5% milk in PBS-Tween20 at 37°C, 1 hour. The membrane was then incubated with either 5 µg/ml biotinylated ARP1, ARP3, VHHR2 or 1∶2000 monoclonal mouse anti-VP6 (AMS Biotechnology, Abingdon, UK) in 5% milk in PBS-Tween20 at 37°C, 1 hour. The membrane was then washed in PBS-Tween20 3 times, 10 minutes/wash and incubated with either 1∶5000 alkaline phosphatase-conjugated streptavidin (Promega, Southampton, UK), for membranes treated with biotinilated primary antibodies or 1∶1000 goat anti-mouse HRP (Dako, Ely, UK), for membranes treated with the mouse monoclonal antibodies, in 5% milk in PBS-Tween20 at 37°C, 1 hour. The membrane was then washed in PBS-Tween20 3 times, 10 minutes/wash. Membranes were developed using the appropriate chemilluminescence reagent, either CDP-Star (Life Technologies, Paisley, UK) for the alkaline phosphatase or ECL Reagent (GE Healthcare, Little Chalfont, UK) for the HRP.

### Immune electron microscopy - Immune capture

Formvar-coated copper grids were coated by floating on a solution of streptavidin (Prozyme, Hayward, CA, USA) (5 µg/ml) in distilled water overnight. Grids were washed by floating on distilled water, blotted and floated onto a 1∶10 or 1∶20 dilution of the biotinylated antibody (ARP1 or control VHH R2). The grid was incubated for 1 hour at 37°C and washed as before. The grids were floated onto 10% suspensions of rotavirus positive faeces or undiluted cell culture supernatants and incubated at 37°C for 1 hour. Grids were washed twice and floated onto 3% phosphotungstic acid (pH 6.3) (Agar Scientific Ltd, Essex, UK) for 1 minute, blotted and allowed to dry in air before examination in the JEOL JEM 1200EX electron microscope. The number of virus particles seen in 5 grid squares was determined and the results expressed as particles/grid square. Specific reactivity was inferred from an increase (≥4 fold) in the number of particles/grid square in the grid coated with ARP1 when compared to the control grid.

## Results

### In vitro neutralising activity of the ARP1 and ARP3 antibody fragments

The initial experiments carried out in The Netherlands with strains Wa, SA11, RRV, or WI61 were performed in CaCo-2 cells, and in MA104 for strain DS-1, and only neutralising activity of ARP1 was determined with VHH R2 as control. In subsequent experiments carried out in the UK, in MA104 cells, both ARP1 and ARP3 rotavirus-specific antibodies were tested in addition to the control VHH R2.

**Figure 1:**
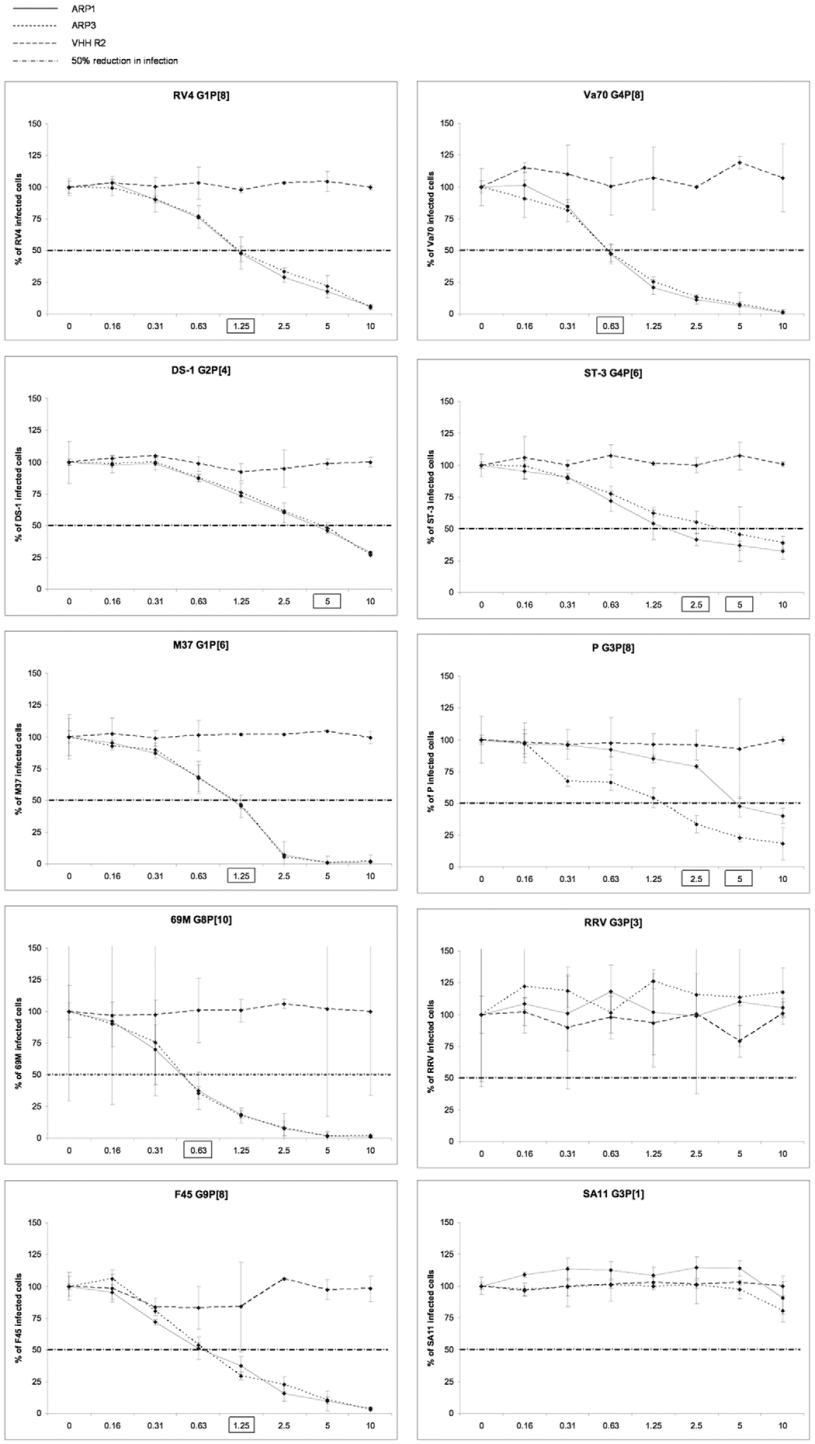
Neutralisation of cell culture adapted rotavirus strains in MA104 cells. Neutralisation experiments were performed using rotavirus-specific antibody fragments ARP1 and ARP3 and control antibody VHH R2. Bars represent percentage of infected cells (fffu) compared with absence of any antibody. A 95% confidence interval is shown by the error bar. A dashed line indicating 50% reduction in fffu is highlighted, and the antibody concentration at which this is achieved is boxed. Concentration of ARP or VHH R2 is expressed in µg/ml on the X-axis in each graph.

The combined results are shown in Table 1. ARP1 showed neutralising activity with all strains but SA11 and one of the RRV strains. ARP3 showed similar neutralising activity to ARP1 with the strains that were tested. The concentration of ARP1 or ARP3 required to give a 50% reduction in fffu was different among strains and ranged from 0.63 µg/ml to 5 µg/ml (Table 1 and Figure 1). No neutralising effect in any of the assays was detected with the control antibody VHH R2.

**Table 1:** Infection neutralisation of different rotavirus strains with rotavirus-specific antibody fragments ARP1 and/or ARP3.

			Infection neutralisation with antibody fragments				
RV strain	Genotype	Cell line	ARP1	conc. for ≥50% reduction fffu	ARP3	conc. for ≥50% reduction fffu	VHH R2
Wa	G1P [8]	CaCo-2	Yes	0.63 µg/ml	NT		No
SA11	G3P [1]	CaCo-2	No		NT		No
RRV (RIVM strain)	G3P [3]	CaCo-2	Yes	2.5 µg/ml	NT		No
WI61	G9P [8]	CaCo-2	Yes	2.5 µg/ml	NT		No
69M	G8P [10]	MA104	Yes	0.63 µg/ml	Yes	0.63 µg/ml	No
F45	G9P [8]	MA104	Yes	0.63 µg/ml	Yes	0.63 µg/ml	No
Va70	G4P [8]	MA104	Yes	0.63 µg/ml	Yes	0.63 µg/ml	No
RV4	G1P [8]	MA104	Yes	1.25 µg/ml	Yes	1.25 µg/ml	No
M37	G1P [6]	MA104	Yes	1.25 µg/ml	Yes	1.25 µg/ml	No
DS1	G2P [4]	MA104	Yes	5.0 µg/ml	Yes	5.0 µg/ml	No
ST-3	G4P [4]	MA104	Yes	5.0 µg/ml	Yes	5.0 µg/ml	No
P	G3P [8]	MA104	Yes	5.0 µg/ml		2.5 µg/ml	No
RRV (KI strain)	G3P [3]	MA104	No		No		No
SA11	G3P [1]	MA104	No		No		No

A summary of neutralisation of different tissue-culture adapted rotavirus strains with ARP1 and/or ARP3, with the concentration of antibody required to achieve a 50% reduction in fffu. VHH R2, a non-related llama antibody fragment, was used as a control.

### Characterization of the binding specificity of the Llama antibody

Immune-EM showed that ARP1 bound rotavirus strains of different genotypes from clinical samples including the recently emerged G8, G10 and G12 strains (Table 2). Binding was also demonstrated for those cell culture adapted rotavirus strains for which ARP1 showed neutralisation activity. Two different strains of RRV were used, whilst one showed binding, the other did not, and these results were in agreement with the inability of the rotavirus-specific antibodies to neutralise one of the RRV strains. No significant binding was observed for strains SA11, P and RV4.

**Table 2:** Immune EM with rotavirus strains of different genotypes from clinical samples or cultured reference rotavirus strains.

	Particles per grid square			
Sample	Genotype	ARP1	VHH R2	Ratio ARP1:VHH R2
Rotavirus from Clinical samples				
Sample 1	G1P [8]	168	12	14
Sample 2	G1P [8]	30	5	6
Sample 3	G1P [8]	91	13	7
Sample 4	G2P [4]	40	<1	40
Sample 5	G3P [8]	1600	30	53.3
Sample 6	G4P [8]	47	<1	47
Sample 7	G9P [6]	217	25	10.8
Sample 8	G9P [8]	74	3	24.7
Sample 9	G12P [9]	77	13	5.9
Sample 11	G10 P [11]	30	2	15
Sample 12	G10 P [11]	116	<1	≥116
Sample 12	G10 P [11]	22	1	22
Cell culture Fluid				
Wa	G1P [8]	330	<1	330
WI61	G9P [8]	11	14	0.78
DS1	G2P [4]	227	3	75.7
RRV (RIVM Strain)	G3P [3]	455	27	16.9
UP3	G9P [6]	12	<1	12
SA11	G3P [1]	2	<1	2
SA11	G3P [1]	5	<1	≥5
RV4	G1P [8]	3	2	1.5
P	G3P [8]	1	4	0.3
ST-3	G4P [6]	82	1	82
Va70	G4P [8]	88	1	88
69M	G8P [10]	6	2	3
F45	G9P [8]	10	<1	≥10
RRV (KI strain)	G3P [3]	5	24	0.2

Two different antibody batches were used, one batch was used at 0.14 mg/ml and the results are shown in italics, the second batch was used at 0.15 mg/ml. The number of virus particles seen in 5 grid squares was determined and the results expressed as particles/grid square. Specific reactivity was inferred from an increase (≥4 fold) in the number of particles/grid square in the grid coated with ARP1 when compared to the control grid.

### Determination of proteins involved in antibody binding by Western blotting

**Figure 2:**
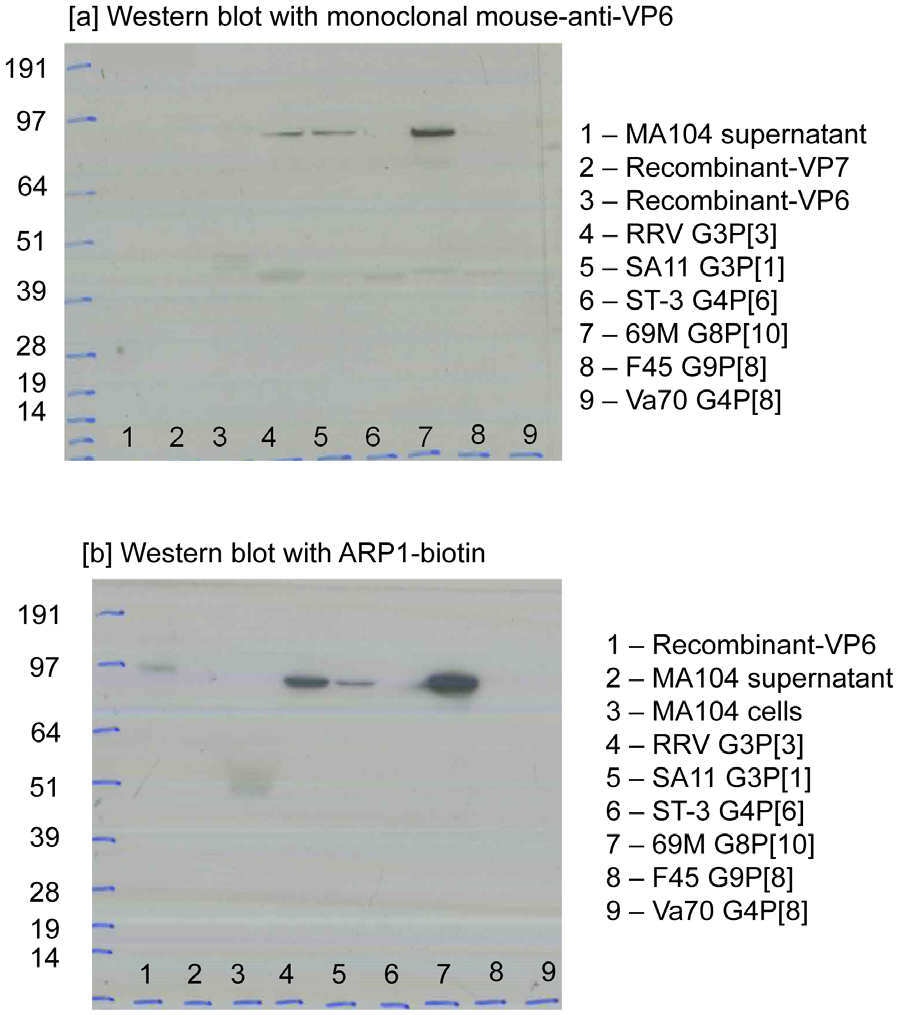
Western blots. Western blots were performed with either [a] monoclonal mouse anti-VP6 or [b] ARP1-biotin. Concentrated cell adapted rotaviruses were run on an SDS-PAGE gel, transferred to a nitrocellulose membrane, and blotted with appropriate antibodies.

In order to identify the viral protein recognised by these antibody fragments, rotavirus strains RRV, SA11, ST-3, 69 M, Va70 and F45, were subjected to SDS-PAGE. Recombinant VP6 and VP7 proteins derived from clinical isolates and expressed in a baculovirus/insect cell system were included as controls. The results showed that both ARP1 and ARP3 recognised bands corresponding to those also recognised by the anti-VP6 monoclonal antibody, representing polymeric VP6, including the recombinant VP6 protein (Figure 2). Neither antibody reacted with any other rotavirus protein, nor did VHH R2 show any reactivity.

## Discussion

Llama antibody fragments ARP1 and ARP3, obtained after immunisation of a llama with RRV G3P [3], were tested for binding and/or neutralising activity towards a broad range of different rotavirus genotypes that were representative of the most common and recently emerged strains worldwide.

Results obtained in the neutralisation assays show that ARP1 neutralised infectivity with rotavirus strains Wa (G1P [8]), DS-1 (G2P [4]), WI-61 (G9P [8]), 69M (G8P [10]), F45 (G9P [8]), Va70 (G4P [8]), RV4 (G1P [8]), M37 (G1P [6]), ST-3 (G4P [4]) and P (G3P [8]), although there were differences in the concentration of antibody required to achieve a 50% reduction in fffu. ARP3 demonstrated similar neutralisation activity to ARP1, with one exception. ARP3 showed greater neutralising activity against strain P than ARP1, and lower concentrations were required to achieve a 50% reduction in fffu. The control antibody did not neutralise any of the tested rotavirus strains, indicating the neutralising activity was specific for ARP1 and ARP3. Neither ARP1 nor ARP3 were able to neutralise SA11. Interestingly, two RRV strains sourced from different laboratories exhibited different binding patterns and neutralisation results. It is unclear whether these differences are due to the differences in the cell line used for virus propagation and neutralisation assays, or whether the strains represent different clones, one of which may have a mutation or mutations in an epitope recognised by these antibody fragments. Previously, Pant *et al* [24] demonstrated neutralising ability of ARP1 both *in vitro* and *in vivo* in a mouse model, and reported that 125 ng/ml of VHH1 (ARP1) was able to show 80% reduction in infection in cell culture using MA104 cells. Detailed characterisation through genome sequencing and comparison of the deduced amino acid sequences of the viral proteins of these two strains may reveal mutations responsible for the observed differences and could also provide some insight into the viral protein that is recognised by these llama antibody fragments.

It has been suggested both in this study, and in other published works, that VP6 is recognised by ARP1, but the rotavirus protein(s) involved viral neutralisation has yet to be fully characterised. Immune EM experiments using EDTA-treated virus particles (to remove the outer layer and expose the VP6) have been inconclusive in elucidating the exact mechanism by which virus neutralisation occurs (data not shown). Previous work has showed that ARP1 and ARP3 recognised different epitopes as they do not compete (H. Marcotte, personal communication). The broad reactivity and neutralising capacity of these antibody fragments indicates that they recognise cross-reactive epitopes. The outer layer proteins VP7 and VP4 both contain neutralising epitopes, but these tend to be type-specific. The middle layer protein, VP6, is the most abundant and immunodominant viral protein, and contains the group and subgroup determining epitopes, and mostly cross-reactive epitopes; for this reason antibodies that recognise this protein are often used in diagnostic immunocapture assays.

Western blots were performed using biotinylated ARP1, ARP3 and VHH R2, and a variety of rotavirus strains that were neutralised by ARP1 and ARP3 as well as the SA11 and RRV strains that were not neutralised. ARP1 and ARP3 recognised the polymeric VP6 of all strains tested, including SA11 and RRV. This suggests that binding may not necessarily correlate with neutralising activity, and that neutralisation may be dependent on the recognition of a conformational epitope which may also be influenced by the adjacent proteins VP7 and VP4, similar to previously reported phenotypic changes for VP4 depending on the VP7 context [25].

The immune-capture experiments indicated that the ARP1 bound to a range of rotavirus genotypes. Interestingly, of the rotavirus strains derived from faecal samples, the antibody was most reactive against G3P [8], whereas reactivity was highest against Wa (G1P [8]) when the cell culture supernatants were examined, when compared against the VHH R2 control. Also, several of the cell culture adapted strains did not show significant reactivity with ARP1 in immune EM although their infectivity was neutralised in cell culture. This apparent contradiction in the results is likely to be due to low viral titres in some of the cell culture supernatants as reflected by the particle counts obtained in the absence of antibodies. Typically, viral loads as high as 10^11^–10^12^ virus particles per gram of faeces are found in clinical samples during the acute phase of disease, whereas typical viral loads achieved in cell culture range from 10^3^–10^7^/ml. For example, for those strains for which the results of the neutralisation and immune-EM assays did not provide a good correlation, the titres were 3.8×10^3^ fffu/ml, 9.1×10^4^ fffu/ml and 2.8×10^5^ fffu/ml for strains RV4, P and 69 M, respectively.

For some time it was thought that antibodies directed against VP6 have no neutralising activity, however, evidence to the contrary has been mounting. Anti-VP6 secretory IgA binds to rotavirus double-layered particles conferring protection by intracellular neutralisation following transcytosis in mice [26], [27], [28]. Recently, llama-derived antibodies that bind specifically to rotavirus VP6 have been shown to neutralise infection with a variety of rotavirus genotypes *in vitro*, and in a neonatal mouse model [24]. The mechanism by which the llama antibodies neutralise infection is not yet understood, but Garaicoechea *et al* speculated that the VP6-specific VHH may block VP6 interaction with a cellular receptor [29], [30] or induce a conformational change which prevents attachment of the virus particle. The neutralising capacity of these antibodies may relate to their phenotypic small size, as bivalent VHH antibodies showed much reduced neutralising activity compared to the monovalent VHH [29].

Currently there is no specific therapy to treat rotavirus disease other than oral or intravenous rehydration solution. However, in the areas of the world where rotavirus disease takes the biggest toll, the use of oral rehydration solution is still disappointingly low, and rotavirus infection is still a major cause of mortality in young infants. Several oral vaccines have been developed recently, and these are highly efficacious in preventing severe disease. However, questions remain about their effectiveness in the poorest regions of the world where oral vaccines often fail to induce protection due to concomitant infections in malnourishment children. In these populations many doses are often required, with concomitant cost and logistical implications, but also, as rotavirus infections occur very early in life; children are exposed to natural rotavirus infection before vaccine-induced protection can be achieved. Recent vaccine trials in Africa and Bangladesh showed that the efficacy of the current live-attenuated vaccines is significantly lower to that observed in developed countries [31], [32]. However, it is considered that even with this reduced efficacy, they are expected to have a major impact in the reducing mortality and SAGE recommends the inclusion of rotavirus vaccination of infants into all national immunization programmes (http://www.who.int/wer/2009/wer8423.pdf).

Several studies have been performed following passive immunisation strategies using bovine colostrum [33], [34] or hyperimmunised chicken egg yolk immunoglobulin [35]. These studies indicated the possible benefits that can be achieved through anti-rotavirus prophylaxis. However, product yield and cost remain the limiting factors. An antiviral chemotherapeutic agent has also been trialled for the treatment of rotavirus diarrhoeal disease, acetorphan (racecadotril). This encephalinase inhibitor has in fact been shown to be effective in reducing the stool output of young children with acute diarrhoea [36]. Finally, probiotics have attracted a renewed interest in last few years, particularly focusing on their effects in treating and preventing diarrheal diseases [37]. However, costs will also remain a limiting factor for the broad use of these promising new developments in developing countries, where they are most needed. As a consequence, there is no satisfactory prophylactic (or therapeutic) means of controlling rotavirus infection, and alternative therapies are still needed. Here we have provided evidence that the two llama-derived antibody fragments, ARP1 and ARP3, have the potential to be used as new antiviral prophylactic or therapeutic products and may provide a valuable complementary prophylactic measure, particularly for those populations in which the efficacy of the live attenuated vaccines is suboptimal. ARP1 has previously been shown to reduce rotavirus-induced diarrhoea in the mouse model [22]. However, only human intervention studies currently under way will determine their usefulness in the prevention or treatment of rotavirus-induced diarrhoeal disease.
